# Cold Bathing

**Published:** 1893-06

**Authors:** 


### COLD BATHING.

Cold bathing in the early morning is beneficial only to those persons who have sufficient vital energy and nervous force to insure good reaction with no subsequent languor or lassitude. Many persons who are greatly refreshed by their morning bath feel tired or languid two or three hours after it. When this occurs it is conclusive evidence against the practice. Persons who have an abundance of blood and flesh, who are lymphatic or sluggish in temperament, and whose nervous force is not depleted, can take the cold morning bath to advantage. Others who are inclined to be thin in flesh, whose hands and feet become cold and clammy on slight provocation, who digest food slowly, and assimilate it with difficulty, who are nervous and who carry large mental burdens, should avoid early morning bathing. For all such the bath at noonday or before retiring at night is far more desirable, and it should be followed by rest of body and brain till equable conditions of circulation are re-established. Some individuals who are weak in nervous power have such excitable peripheral nerves that they get at once a perfect reaction from cold bathing, but lose in after effects more than the value of the bath. This class of persons should not bathe too often, and should always use tepid water, choosing the time preferably before retiring.

